# External Oblique Intercostal Plane Block Versus Port-Site Infiltration for Laparoscopic Sleeve Gastrectomy: A Randomized Controlled Study

**DOI:** 10.1007/s11695-024-07219-z

**Published:** 2024-04-02

**Authors:** Omer Doymus, Ali Ahiskalioglu, Ahmet Kaciroglu, Zehra Bedir, Serkan Tayar, Mustafa Yeni, Erdem Karadeniz

**Affiliations:** 1 Department of Anaesthesiology and Reanimation, Erzurum Regional Training and Research Hospital, Erzurum, Turkey; 2 Department of Anaesthesiology and Reanimation, Ataturk University School of Medicine, 25070 Erzurum, Turkey; 3 Clinical Research, Development and Design Application and Research Center, Ataturk University School of Medicine, 25240 Erzurum, Turkey; 4 Department of Anaesthesiology and Reanimation, Bursa City Hospital, Bursa, Turkey; 5 Department of General Surgery, Erzurum Regional Training and Research Hospital, Erzurum, Turkey; 6 Department of General Surgery, Ataturk University School of Medicine, Erzurum, Turkey

**Keywords:** External oblique intercostal block, Obesity surgery, Pain, Port-site infiltration

## Abstract

**Purpose:**

Although laparoscopic sleeve gastrectomy (LSG) is a minimally invasive surgery, postoperative pain is common. A novel block, the external oblique intercostal (EOI) block, can be used as part of multimodal analgesia for upper abdominal surgeries. The aim of our study is to investigate the effectiveness of EOI block in patients undergoing LSG.

**Materials and Methods:**

Sixty patients were assigned into two groups either EOI or port-site infiltration (PSI). The EOI group received ultrasound-guided 30 ml 0.25% bupivacaine, while the PSI group received 5 ml of 0.25% bupivacaine at each port sites by the surgeon. Data on clinical and demographic were collected and analyzed.

**Results:**

There were no statistical differences in terms of demographic details (*p* > 0.05). VAS scores were statistically lower during resting at PACU, 1, 2, 4, 8, and 12 h postoperatively in the EOI group than PSI group (*p* < 0.05), The VAS scores were also lower during active movement at PACU, 1, 2, 4, and 8 h postoperatively in the EOI group than PSI group (*p* < 0.05). Twenty-four-hour fentanyl consumption was lower in the EOI than in the PSI group (505.83 ± 178.56 vs. 880.83 ± 256.78 μg, respectively, *p* < 0.001). Rescue analgesia was higher in PSI group than EOI group (26/30 vs. 14/30, respectively, *p* = 0.001).

**Conclusion:**

EOI block can be used as a part of multimodal analgesia due to its simplicity and effective postoperative analgesia in LSG.

**Graphical Abstract:**

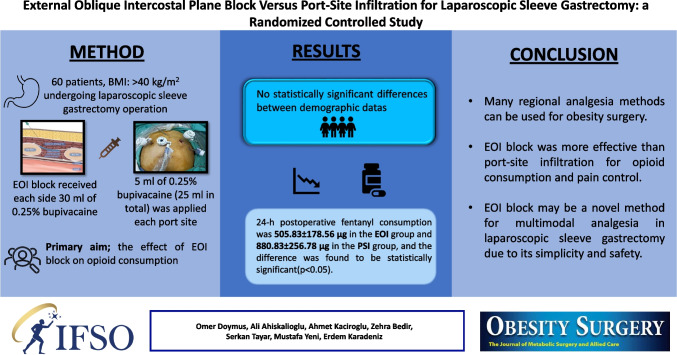

## Introduction

Although obesity is associated with increased morbidity and mortality, lifestyle changes that lead to short-term weight loss can enhance overall health. Bariatric surgery is the most effective treatment for weight loss, reducing obesity-related comorbidities and mortality. This surgery is improving quality of life [1].

The laparoscopic sleeve gastrectomy (LSG) has been shown to be related to lower complication rates, shorter hospital stay, and earlier re-engagement in normal activities than open procedures [2]. LSG is performed through small incisions in the abdominal wall. However, postoperative pain is common. Pain often originates from port sites in the abdominal wall [3]. Uncontrolled pain may cause early ambulation and delay in performing deep breathing exercises in patients with obstructive sleep apnea and cardiac comorbidities. This increases the risk of deep vein thrombosis and pulmonary complications [4, 5].

Obesity is associated with anatomical and pathophysiologic pharyngeal abnormalities that predispose to airway collapse. Systemic opioids suppress respiratory system, supraglottic airway muscle tone, and level of consciousness. Therefore, hypoxia and hypercapnia develop. The effects of opioid use and obesity-induced ventilatory impairment greatly increase the risk of pulmonary complications. Therefore, it is wise to use opioid-sparing or opioid-free analgesia for perioperative analgesia [6].

Agents such as non-steroidal anti-inflammatory drugs, paracetamol, tramadol, gabapentinoids, dexmedetomidine, and ketamine, intravenous lidocaine can be used for analgesia in bariatric surgery. However the use of these agents after bariatric surgery is generally limited [6–8].

Although epidural anesthesia is effective in pain control, positioning is extremely difficult in obese patients. The use of regional anesthesia techniques such as transversus abdominis plane block and erector spina plan block provides fewer opioid use and better pain management [9–11].

External oblique intercostal (EOI) block is one of the novel interfascial plane block. EOI provides dermatomal sensory blockade involving T6–T10 in the anterior axillary line and T6-T9 in the midline. EOI can be performed as part of multimodal analgesia for upper abdominal surgeries [12]. In addition, easy visualization of the application area with USG is an advantage for obese patients [13]. In the literature, studies on the analgesic efficacy of EOI block in bariatric surgery are still limited.

The primary aim of this study was to investigate the effect of EOI block on opioid consumption, and the secondary aim was to investigate the effect on pain scores in patients undergoing LSG.

### Material Method

In this prospective randomized controlled study, after ethics committee approval (Ataturk University, Erzurum, Turkey, 27.01.2022-B.30.2.ATA.0.01.00/106, ClinicalTrials.gov NCT05614921), a total of 60 participants, ASA II–III group, aged 18–60 years, having a BMI > 40 kg/m^2^, and undergoing laparoscopic sleeve gastrectomy operation, were included. Patients who did not want to participate in the study, patients with serious underlying cardiovascular disease, patients with liver dysfunction, patients with coagulopathy or on anticoagulant drugs, patients who were unable to cooperate, and patients who were allergic to one of the drugs to be used were excluded.

Patients were randomly divided into two equal groups using Microsoft Excel RAND function to receive either an EOI block or port site infiltration. Prior to being transferred to the operation room, all patients received pantoprazole 40 mg and metoclopramide 10 mg in the ward.

The same general anesthesia protocol was applied to all patients. Routine monitoring including SO_2_, heart rate, and noninvasive arterial blood pressure was performed after the patients were admitted to the operating room.

#### Port Site Infiltration Group

After the patient was orotracheally intubated, port entry sites were determined. A total of 5 ports were inserted: one port (10 mm, cutting) 5 cm above the umbilicus on the linea alba (camera port), two ports (12 mm, blunt) approximately 8 cm lateral to the camera port in the right and left upper quadrants, one port (5 mm, blunt) in the subxiphoid region, and one port (5 mm, blunt) approximately 12 cm distal to the camera port on the left side at the intersection of the posterior axillary line and the umbilicus. After the port entry sites were determined and marked, with a 21-gauge needle, 5 ml of 0.25% bupivacaine (25 ml in total) was applied under the aponeurotic layer at each port entry site by the surgeon.

#### External Oblique Intercostal Plane Block Group

**Fig. 1:**
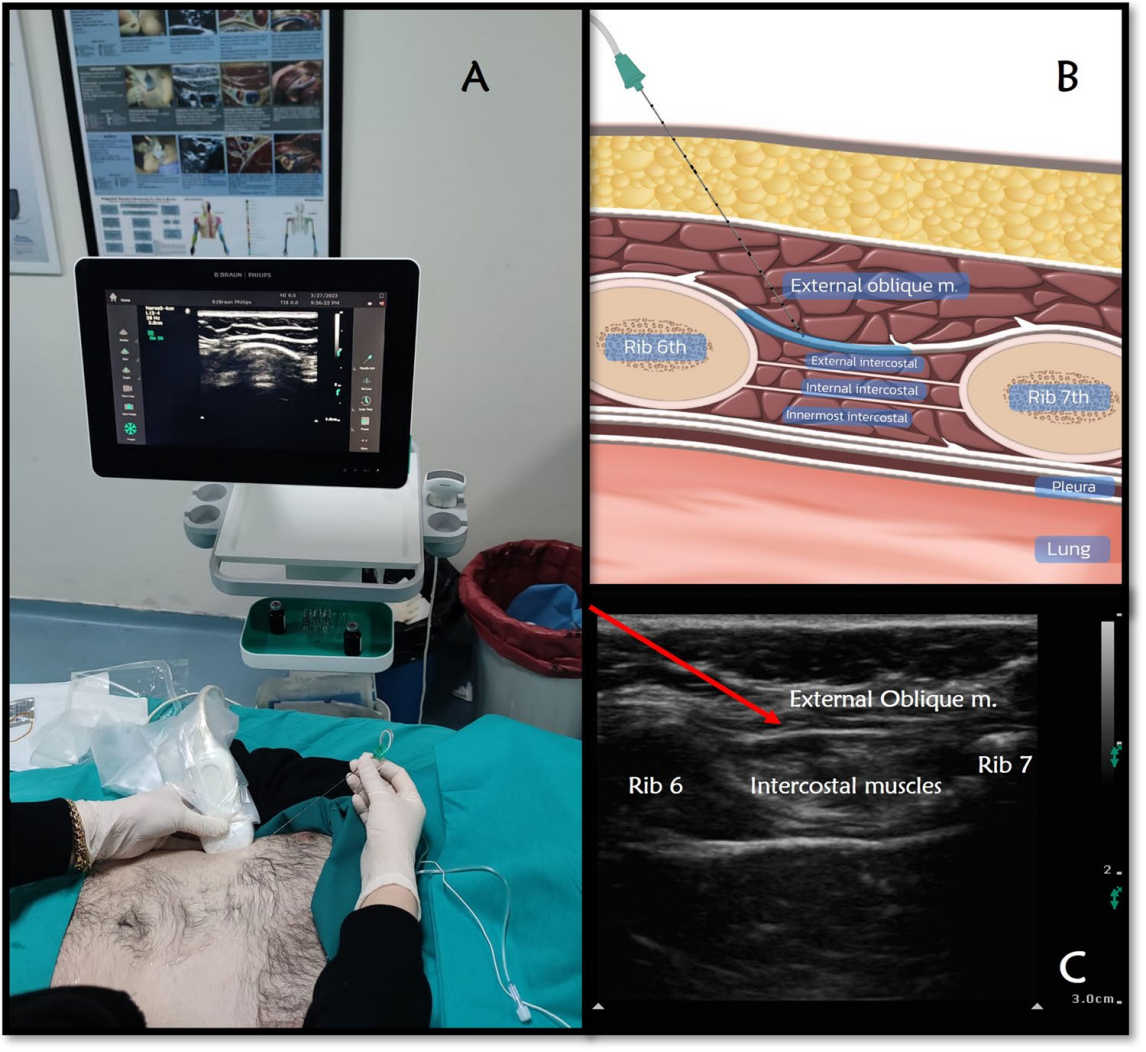
A Patient and ultrasound set up for External oblique intercostal (EOI) block B Basic illustration of the EOI block. C Sono-anatomic structures of EOI block. Red arrow; needle trajectory

The patient was taken to the regional anesthesia room 45 min before the start of surgery and monitored. In supine position, the area to be treated and the linear USG probe were prepared sterile. After the 12–15 Hz linear ultrasound transducer was placed obliquely medial to the anterior axillary line, the 6th and 7th ribs, skin, subcutaneous tissues, and external oblique muscle were identified (Fig. 1). The needle was directed under the external muscle with in-plane technique. After 2 ml of saline was used to confirm the location of the needle, for each side, 30 ml of 0.25% bupivacaine was applied between the external oblique muscle and intercostal muscles.

The 10-mm trocar entry site, which was employed as the camera entry site, was closed with fascia in each group. The closure process was not implemented for the remaining items.

#### Postoperative Analgesia

Paracetamol 1000 mg IV was given to each patient 30 min before the end of surgery and repeated every 6 h in the postoperative period. The same protocol was applied for postoperative analgesia in both groups. Patients were extubated and taken to the PACU. For postoperative analgesia, a patient-controlled analgesia device (PCA) was implanted iv in the PACU. The PCA device prepared with fentanyl was programmed with a concentration of 10 mcq/ml, 15 min locked time, 25 mcq bolus, and no basal infusion and continued for 24 h. In the recovery room, 25 mg meperidine was administered additionally to patients with a VAS score of 4 and above and recorded. Patients with an Aldrete score of 9 and above were transferred to the ward. Postoperative follow-up and evaluation of the patients were performed by an investigator who was not informed about the study groups. Postoperative pain assessment was performed base on the visual analog scale at 1, 2, 4, 8, 12, and 24 h.

### Statistical Analysis

To decide on the required sample size, a pilot study has been done. The pilot study showed that our primary aim—24-h opioid consumption (fentanyl-mcq)—should be 550.00 ± 111.80 mcq in the EOI group (*n* = 8) and 743.75 ± 244.86 mcg in the port-site group (*n* = 8). A sample size of 27 patients in total was computed for each group via G*Power version 3.1.9.2 (Heinrich Heine University Düsseldorf) with an effect size of 1.017, a power of 0.95, and an alpha probability of 0.05. Considering dropouts, it was decided that at least 60 participants would be recruited. Data was analyzed using SPSS Statistics 22 software (IBM, Armonk, New York, USA). Following assessment for normal distribution with the Kolmogorov–Smirnov test, the normal distributing data were analyzed with Student’s *t*-test, and non-normally distributed data were evaluated using the Mann–Whitney *U* test. Categorical data such as the need for rescue analgesic, complications, and adverse events were assessed using chi-square tests and Mann–Whitney *U* or Student’s *t*-tests for continuous measures. Statistical significance was accepted when *p* < 0.05. All *p* values were calculated as two-sided.

## Results

**Fig. 2:**
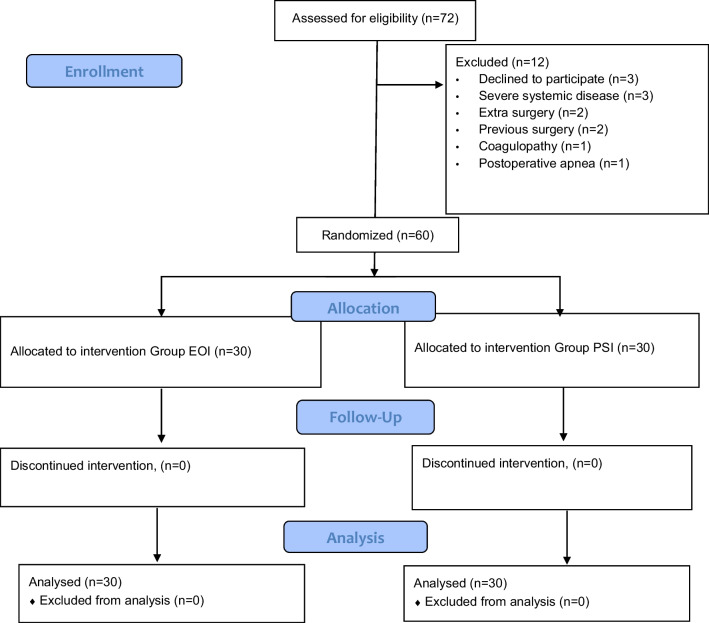
CONSORT flow diagram of the study

After excluding 12 out of the 72 eligible patients, a total of 60 patients were randomly assigned to two different groups (Fig. 2). The patient age was 39.17 ± 11.60 years in the EOI group and 37.80 ± 13.17 years in the PSI group. The patient weight was 124.57 ± 19.63 kg in the EOI group and 128.43 ± 22.26 kg in the PSI group. There were no statistically significant differences between the groups in terms of weight, age, height, BMI or the duration of the surgery (*p* > 0.05). Detailed results were reported in Table 1.

**Table 1:** Comparison of demographic data

	Group EOI (*n* = 30)	Group PSI (*n* = 30)	*p*
Age(years)	39.17 ± 11.60	37.80 ± 13.17	0.636^a^
Weight(kg)	124.57 ± 19.63	128.43 ± 22.26	0.477^a^
Height (cm)	163.43 ± 8.32	165.03 ± 9.77	0.630^a^
BMI (kg/m^2^)	46.52 ± 3.30	47.04 ± 6.04	0.464^a^
Duration of the surgery	82.33 ± 18.93	81.67 ± 18.07	0.976^a^
Gender (F/M)	22/8	21/9	0.774^b^

Values are expressed as mean ± standard deviation or number. *BMI* body mass index

^a^Mann-Whitney *U* test between groups

^b^Chi-square test between groups

The VAS scores were also significantly higher during resting at PACU, 1, 2, 4, 8, and 12 h postoperatively in the PSI group than in the EOI group (*p* < 0.05), resting vas score was similar in both groups at 24 h (*p* > 0.05). The VAS scores were also significantly higher during active movement at PACU, 1, 2, 4, and 8 h postoperatively in the PSI group than in the EOI group (*p* < 0.05). VAS score in active movement were similar in both groups at 12 and 24 h (*p* > 0.05) (Table 2 and 3).

**Table 2:** VAS scores in resting

	Group EOI (*n* = 30)	Group PSI (*n* = 30)	*p*
PACU	4.03 ± 3.48	7.83 ± 2.46	** < 0.001**^**a**^
1st hour	3.83 ± 3.17	6.87 ± 2.52	**0.001**^**a**^
2nd hour	2.83 ± 2.79	5.30 ± 2.87	**0.002**^**a**^
4th hour	2.17 ± 2.20	4.07 ± 2.26	**0.002**^**a**^
8th hour	2.17 ± 2.04	3.47 ± 1.98	**0.011**^**a**^
12th hour	1.93 ± 2.07	3.03 ± 2.17	**0.031**^**a**^
24th hour	1.67 ± 1.88	2.60 ± 2.49	0.091^a^

Values are expressed as mean ± standard deviation. *PACU* postanesthetic care unit

^a^Mann-Whitney *U* test between groups

**Table 3:** VAS scores in active movement

	Group EOI (*n* = 30)	Group PSI (*n* = 30)	*p*
PACU	5.37 ± 3.33	8.90 ± 1.94	** < 0.001**^**a**^
1st hour	5.57 ± 2.81	7.83 ± 2.64	**0.002**^**a**^
2nd hour	4.23 ± 2.54	6.13 ± 2.91	**0.012**^**a**^
4th hour	3.70 ± 2.42	5.03 ± 2.51	**0.038**^**a**^
8th hour	3.30 ± 2.59	4.57 ± 2.18	**0.028**^**a**^
12th hour	3.00 ± 2.39	4.07 ± 2.33	0.084^a^
24th hour	2.73 ± 2.48	3.53 ± 2.36	0.158^a^

Values are expressed as mean ± standard deviation. *PACU* postanesthetic care unit

^a^Mann-Whitney *U* test between groups

The 24-h postoperative fentanyl consumption was 505.83 ± 178.56 µg in the EOI group and 880.83 ± 256.78 µg in the PSI group, and the difference was found to be statistically significant (*p* < 0.001). The number of patients requiring rescue analgesia was 14 in the EOI group and 26 in the PSI group (*p* = 0.001) (Table 4). No statistically significant differences between the groups were observed in terms of side effects (*p* > 0.05) (Table 5).

**Table 4:** Fentanyl consumption (µg) via patient-controlled analgesia

	Group EOI (*n* = 30)	Group PSI (*n* = 30)	*p*
0 to 4 h (µg)	158.33 ± 70.51	250.00 ± 90.73	< 0.001^a^
4 to 8 h (µg)	161.67 ± 93.25	250.00 ± 105.45	0.001^a^
8 to 24 h (µg)	178.33 ± 88.99	380.83 ± 157.80	< 0.001^a^
24 h total (µg)	505.83 ± 178.56	880.83 ± 256.78	< 0.001^a^

Values are expressed as mean ± standard deviation

^a^Mann-Whitney *U* test between groups

**Table 5:** Need for rescue analgesics and side effects

	Group EOI (*n* = 30)	Group PSI (*n* = 30)	*p*
Need for rescue analgesia	14	26	0.001^b^
Nausea	6	6	1.000^b^
Vomiting	1	2	0.554^b^
Need for antiemetics	2	2	1.000^b^
Constipation	0	0	NS
Itching	1	0	0.313^b^
Urinary retention	0	0	NS
Dry mouth	1	5	0.085^b^
Block-related complication	0	0	NS

Values are expressed as mean ± standard deviation. *NS* non-significant

^b^Chi-square test between groups

## Discussion

In this study, EOI block reduced postoperative opioid consumption and improved postoperative pain levels in patients after LSG surgery compared to the port site infiltration.

The main purpose of the multimodal management of analgesia to pain in bariatric surgery is to minimize opioid consumption or to avoid opioids as much as possible [14]. The main interest in opioid-sparing analgesic methods for patients with morbid obesity has been driven primarily by an effort to raise the safety of acute pain management [15]. Although opioids are effective in suppressing hormonal stress responses induced by surgery and reducing hemodynamic imbalance, they may cause unwanted side effects such as hyperalgesia, vomiting, nausea, and respiratory depression. In addition, opioid-based anesthesia has been reported to contribute to a reduced risk of persistent postoperative pain. For these reasons, clinical efforts are being made to develop opioid-free and opioid-sparing anesthesia strategies [16–18].

There is a theoretical risk of gastrointestinal (GI) ulceration and surgical bleeding with NSAIDs, and routine use is usually avoided after bariatric surgery [19]. Preperitoneal local anesthesia with bupivacaine has been shown to lead to a decline in opioid consumption and postoperative pain during mobilization, at rest and 6 h after surgery, and this procedure has been shown to reduce the incidence of chronic postoperative pain after laparoscopic bariatric surgery [20].

Epidural anesthesia has been associated with reduced pulmonary complications and postoperative opioid requirements in patients with morbid obesity undergoing open abdominal surgery and thoracic surgery [21]. In addition, it has been reported that local anesthetic spread in the epidural space is high level in patients with morbid obesity due to narrowing of the epidural space [22]. Epidural anesthesia is associated with risks of potential neurological complications, epidural-related infection, placement failure, and other technical complications, which are relatively common in the obese population due to epidural placement failure, difficulty in identifying anatomical landmarks, and the need for longer needles. Catheter dysfunction requiring repositioning even after a successful epidural due to excessive mobility of the overlying soft tissue is more common in obese patients [23, 24].

The anterior branches of the T6–T12 nerves continue as intercostal nerves between the internal oblique muscles and transversus abdominis muscle. The nerve branches form lateral cutaneous branches that innervate the lateral abdominal wall and the mid-axillary line at the junction of the external oblique muscle and serratus anterior muscle. The lateral cutaneous branches at the midaxillary level can be blocked by applying local anesthetic to the external oblique intercostal plane between the anterior and middle axillary line at the level of the sixth or seventh rib. The external oblique muscle fascia participates with other muscles’ fascia to form the anterior rectus sheath. Local anesthetics follow this pathway and reach the rectus sheath, which is the entry point of the terminal anterior cutaneous branches of the thoracoabdominal nerves and block the cutaneous branches of the relevant intercostal nerves in the anterior region. Thus, in EOI block, the lateral and anterior branches of the T6–T10 intercostal nerves between the fascial layers are blocked, and analgesia is achieved.

EOI block represents a novel alternative approach to regional anesthesia involving neuraxial or deep plane blocks and patient-controlled opioid analgesia in upper abdominal surgery [25–27]. The EOI plane can be defined superficially and rapidly even in obese patients. Some of the advantages of the EOI block are that it is applied in the supine position, it is more superficial compared to ESP at the T7 level, and the needle/catheter entry site is far from the surgical site. In addition, like ESP and transmuscular quadratus lumborum block, it can provide analgesia in the T7–T11 dermatomes of the lateral and anterior abdominal wall [12, 27]. One of the limitations of the EOI block, like other fascial plane blocks, is the lack of visceral analgesic coverage [28]. The EOI block also does not consistently extend below the umbilicus. The fact that the application site can be easily visualized and easily accessible regardless of body mass index is also not a disadvantage for obese patients under USG.

Çoşarcan et al. administered an EOI block using a 20 ml solution of 0.25% bupivacaine, along with TAP and rectus sheath blocks, in different upper abdominal procedures [13]. In a cadaveric study, Elsharkawy et al. showed that both the anterior and lateral branches of the T7–T10 intercostal nerves were stained in EOI block with 29 mL of 0.25% bupivacaine and 1 mL of India ink. Additionally, they demonstrated that sensory blockage was successfully achieved in the T6–T10 dermatomes along the anterior axillary line and T6–T9 dermatomes along the midline [12]. We administered a substantial amount of local anesthetic since we believed that more analgesia may be attained by utilizing a larger volume for fascial plane blocks, drawing from prior research findings and our previous experiences [29, 30].

The results of TAP block are not always encouraging [31]. The lateral cutaneous branches of the intercostal nerves, which lend to the innervation of the upper abdominal wall, are not reliably blocked by TAP block approaches, including subcostal TAP block. Even variants of subcostal TAP block fail to provide analgesia of abdominal region.

Ultrasound-guided bilateral ESPB has been found to increase the need for intraoperative and postoperative opioids in patients with the morbid obesity undergoing bariatric surgery [32]. Even though the implementation area of ESPB is far from the epidural space, epidural-like effects may occur in abdominal surgery [33]. Although it can be performed in lateral decubitus and prone positions under general anesthesia, the difficulty of positioning, especially in obesity patients, challenges clinicians [34]. Despite the technical challenges in ESP block in morbid obesity patients, in our study, we easily performed EOI block in the supine position and using a linear probe at a depth of approximately 2–3 cm. The EOI block, in this configuration, presents itself as a convenient alternative to both paraspinal region and other abdominal wall blocks, owing to its technical simplicity and efficacy.

This study has some limitations. Initially, the authors were unable to assess the detailed dermatome region in patients following the application of EOI block because to time limitations. Furthermore, pain levels were assessed within the initial 24-h period. In one of our limitations despite different port sizes (8–12 mm), the same volume of local anesthetic was injected into all sites. Lastly, the sample size was determined by postoperative opioid consumption, which may not have been adequate for assessing side effects, block-related complications, and pain scores. However, since there is no study on EOI block in LSG surgery, this study will make an essential contribution to the existing literature.

## Conclusion

In conclusion, the main advantages of the EOI block are performing in the supine position, having a distant needle/catheter entry site from the operation area, and being superficially and easily defined with ultrasound, especially in obese patients. EOI plane block could serve as a viable option for regional anesthesia in laparoscopic bariatric surgery due to its convenient, secure, and efficient analgesic properties.

## References

[1] Sjöström L, Lindroos AK, Peltonen M (2004). Lifestyle, diabetes, and cardiovascular risk factors 10 years after bariatric surgery. N Engl J Med.

[2] Ruiz-Tovar J, Muñoz JL, Gonzalez J (2017). Postoperative pain after laparoscopic sleeve gastrectomy: comparison of three analgesic schemes (isolated intravenous analgesia, epidural analgesia associated with intravenous analgesia and port-sites infiltration with bupivacaine associated with intravenous analgesia). Surg Endosc.

[3] Mittal T, Dey A, Siddhartha R (2018). Efficacy of ultrasound-guided transversus abdominis plane (TAP) block for postoperative analgesia in laparoscopic gastric sleeve resection: a randomized single blinded case control study. Surg Endosc.

[4] Idra S, Cristina M, Punzo G (2008). Postoperative analgesia in laparoscopic bariatric surgery. Eur J Anaesthesiol.

[5] Sarandan M, Guragata-Balasa C, Papurica M (2011). Anesthesia in laparoscopic bariatric surgery (gastric sleeve) - preliminary experience. Timisoara Med J.

[6] Macintyre PE, Loadsman JA, Scott DA (2011). Opioids, ventilation and acute pain management. Anaesth Intensive Care.

[7] Miller AD, Smith KM (2006). Medication and nutrient administration considerations after bariatric surgery. Am J Health Syst Pharm.

[8] Sinclair DR, Chung F, Mezei G (1999). Can postoperative nausea and vomiting be predicted?. Anesthesiology.

[9] Ibrahim M, Elnabtity AM, Hegab A (2022). Combined opioid free and loco-regional anaesthesia enhances the quality of recovery in sleeve gastrectomy done under ERAS protocol: a randomized controlled trial. BMC Anesthesiol.

[10] Földi M, Soós A, Hegyi P (2021). Transversus abdominis plane block appears to be effective and safe as a part of multimodal analgesia in bariatric surgery: a meta-analysis and systematic review of randomized controlled trials. Obes Surg.

[11] Abdelhamid BM, Khaled D, Mansour MA (2020). Comparison between the ultrasound-guided erector spinae block and the subcostal approach to the transversus abdominis plane block in obese patients undergoing sleeve gastrectomy: a randomized controlled trial. Minerva Anestesiol.

[12] Elsharkawy H, Kolli S, Soliman LM (2021). The external oblique intercostal block: anatomic evaluation and case series. Pain Med.

[13] Coşarcan SK, Erçelen Ö (2022). The analgesic contribution of external oblique intercostal block: case reports of 3 different surgeries and 3 spectacular effects. Medicine (Baltimore).

[14] Schumann R (2011). Anaesthesia for bariatric surgery. Best Pract Res Clin Anaesthesiol.

[15] Belcaid I, Eipe N (2019). Perioperative pain management in morbid obesity. Drugs.

[16] Hung KC, Chiu CC, Hsu CW (2022). Impact of opioid-free anesthesia on analgesia and recovery following bariatric surgery: a meta-analysis of randomized controlled studies. Obes Surg.

[17] Olausson A, Svensson CJ, Andréll P (2022). Total opioid-free general anaesthesia can improve postoperative outcomes after surgery, without evidence of adverse effects on patient safety and pain management: a systematic review and meta-analysis. Acta Anaesthesiol Scand.

[18] De Cassai A, Geraldini F, Tulgar S (2022). Opioid-free anesthesia in oncologic surgery: the rules of the game. J Anesth Analg Crit Care.

[19] Klein M, Støckel M, Rosenberg J (2012). Intraoperative ketorolac and bleeding after laparoscopic Roux-en-Y gastric by-pass surgery. Acta Chir Belg.

[20] Boerboom SL, de Haes A, VdWetering L (2018). Preperitoneal bupivacaine infiltration reduces postoperative opioid consumption, acute pain, and chronic postsurgical pain after bariatric surgery: a randomized controlled trial. Obes Surg.

[21] Sharma M, Mehta Y, Sawhney R (2010). Thoracic epidural analgesia in obese patients with body mass index of more than 30 kg/m2 for off pump coronary artery bypass surgery. Ann Card Anaesth.

[22] Panni MK, Columb MO (2006). Obese parturients have lower epidural local anaesthetic requirements for analgesia in labour. Br J Anaesth.

[23] Buckley FP, Robinson NB, Simonowitz DA (1983). Anaesthesia in the morbidly obese. A comparison of anaesthetic and analgesic regimens for upper abdominal surgery. Anaesthesia.

[24] Hood DD, Dewan DM (1993). Anesthetic and obstetric outcome in morbidly obese parturients. Anesthesiology.

[25] Erskine RN, White L (2022). A review of the external oblique intercostal plane block - a novel approach to analgesia for upper abdominal surgery. J Clin Anesth.

[26] Korkusuz M, Basaran B, Et T (2023). Bilateral external oblique intercostal plane block (EOIPB) in patients undergoing laparoscopic cholecystectomy: a randomized controlled trial. Saudi Med J.

[27] White L, Ji A (2022). External oblique intercostal plane block for upper abdominal surgery: use in obese patients. Br J Anaesth.

[28] Elsharkawy H, El-Boghdadly K, Barrington M (2019). Quadratus lumborum block: anatomical concepts, mechanisms, and techniques. Anesthesiology.

[29] Ahiskalioglu A, Yayik AM, Celik EC (2022). The shining star of the last decade in regional anesthesia part-i: interfascial plane blocks for breast, thoracic, and orthopedic surgery. Eurasian J Med.

[30] Celik M, Tulgar S, Ahiskalioglu A (2019). Is high volume lumbar erector spinae plane block an alternative to transforaminal epidural injection? Evaluation with MRI. Reg Anesth Pain Med.

[31] Mongelli F, Marengo M, Bertoni MV (2023). Laparoscopic-assisted transversus abdominis plane (TAP) block versus port-site infiltration with local anesthetics in bariatric surgery: a double-blind randomized controlled trial. Obes Surg.

[32] Zengin SU, Ergun MO, Gunal O (2021). Effect of ultrasound-guided erector spinae plane block on postoperative pain and intraoperative opioid consumption in bariatric surgery. Obes Surg.

[33] Pak A, Singh P (2020). Epidural-like effects with bilateral erector spinae plane catheters after abdominal surgery: a case report. A A Pract.

[34] Toprak H, Başaran B, Toprak ŞS, Et T (2023). Efficacy of the erector spinae plane block for quality of recovery in bariatric surgery: a randomized controlled trial. Obes Surg.

